# 

**Published:** 1808-07

**Authors:** 


					THE
<? * 'Kf il
i *
Av^ * '
M E D I OA L
AND
PHYSICAL
?JO II M JV A L,
CONDUCTED BY
T. BRADLEY, M. D.
AND
J. ADAMS, M. D,
VOL. XX.
From June to December, 180S,
l S' S7Z6
iDom
LON
PllISTEP FOR RICIIARD PHILLIPS, NO. 6, BRIDG E-STRKl
ELACKFRIAIIS.
By VVit'.iarr. Thome, Red Lion Court, F>?; Street.
[ sfnteren at ?>tatfoner$ 3
(
NEWcMEDTCAl PUBLICATIONS.
The Vaccine Phantasmagoria, 2s/
The Vaccine Scourge,' J\fo. I. is. 1 ' 1 1
Important Hesearches upon the'Existence, Nature, and Communication
of Venereal Infection in Prfgijant Wometyntfw born Infants, and Nurses^
, by tlie late P? A. 0. Mahon.

				

